# The abnormal distribution of peripheral B1 cells and transition B cells in patients with idiopathic dilated cardiomyopathy: a pilot study

**DOI:** 10.1186/s12872-022-02461-8

**Published:** 2022-03-04

**Authors:** Quan Tang, Zhihong Cen, Jing Lu, Jingwei Dong, Lin Qin, Feiyu Lu, Weifeng Wu

**Affiliations:** 1 Department of Cardiology, First Affiliated Hospital of Guangxi Medical University, Guangxi Cardiovascular Institute, Shuangyong Road 6, Nanning, 530021 Guangxi People’s Republic of China; 2 Department of Cardiology, Fifth Affiliated Hospital of Guangxi Medical University, Nanning, 530022 Guangxi People’s Republic of China

**Keywords:** Dilated cardiomyopathy, Autoimmunity, Flow cytometry, B cell subsets, B1 cells, Transition B cells

## Abstract

**Background:**

The aberrant distribution of peripheral B cell subsets is associated with the pathogenesis of a variety of inflammatory and autoimmune diseases. However, the distribution of peripheral B cell subsets in patients with idiopathic dilated cardiomyopathy (DCM) remains to be elucidated.

**Methods:**

Twenty-seven patients with idiopathic DCM (DCM group), 18 control patients with heart failure (HF group) and 21 healthy individuals (HC group) were included in this study. Peripheral B cell subsets were analysed using multicolour flow cytometry. The plasma β1 adrenergic receptor (β1-AR) autoantibody titre was determined using ELISA. Additionally, clinical features were also collected.

**Results:**

Compared with the HF and HC groups, the percentage of B1 cells was significantly decreased, whereas the percentage of transitional B cells (Tr) was significantly increased in the DCM group. Notably, the percentage of B1 cells was significantly lower in patients with β1-AR autoantibody-positive DCM than in β1-AR autoantibody-negative patients. The correlation analysis showed that the percentage of B1 cells was negatively correlated with N-terminal pro-brain natriuretic peptide (NT-proBNP) levels and positively correlated with the left ventricular ejection fraction in patients with DCM.

**Conclusion:**

As shown in the present study, the percentage of B1 cells in the peripheral blood of patients with idiopathic DCM is abnormally decreased, especially in β1-AR autoantibody-positive patients, while the percentage of Tr cells is significantly increased, indicating that B1 cells and Tr cells may be implicated in the pathogenesis of idiopathic DCM. The decrease in the percentage of B1 cells is directly related to the severity of DCM.

**Supplementary Information:**

The online version contains supplementary material available at 10.1186/s12872-022-02461-8.

## Background

Dilated cardiomyopathy (DCM) is a progressive myocardial disease that is characterized by dilation and contraction dysfunction of the left ventricle or both ventricles without pressure or volume overload or coronary heart disease [1]. The most common complications of DCM are heart failure and malignant arrhythmia, which are the main causes of death [2]. Although the aetiology of DCM is heterogeneous and the pathogenesis is complex and still largely unknown, accumulating evidence indicates that chronic inflammation and autoimmune disorders are closely associated with the pathogenesis of DCM [3–5].

As an essential immune regulator, B lymphocytes play a crucial role in maintaining immune homeostasis and preventing autoimmune diseases. Abnormal numbers or dysfunction of B cells is closely involved in the disorder of autoimmune regulation in patients with DCM. Early research has confirmed that the tolerance of peripheral B cells is impaired, leading to the production of anti-heart autoantibodies, including autoantibodies against the β1 adrenergic receptor (β1-AR), which mediate cardiomyocyte injury and contractile dysfunction [5] and contribute to the pathogenesis of DCM [6]. According to a recent study, the use of CD20 chimeric monoclonal antibodies to deplete peripheral CD20^+^ B cells in patients with DCM reduces the infiltration of T cells and macrophages in the myocardium and significantly improves cardiac function [7].

The peripheral blood circulation is the bridge between the migration of B cells in the bone marrow and the secondary lymphoid organ. A variety of B cell subpopulations are detected in peripheral blood and have their own unique immune functions. As innate immune cells, B1 cells spontaneously secrete low-affinity, multireactive natural IgM antibodies to protect against pathogenic microorganisms in the early stage of infection [8]. In addition, these natural IgM antibodies also accelerate the elimination of apoptotic cell debris and harmful molecules, induce and inhibit the inflammatory signalling cascade in the cell, and may have a function similar to homeostasis [9–13]. Naïve B cells are differentiated from transitional B cells (Tr) and mainly secrete interleukin-10 (IL-10). Memory B cells are an important part of humoral immunity and the main source of inflammatory factors that cause autoimmune diseases [14]. Plasmablasts (PB), which account for less than 1% of circulating B cells, are immature plasma cell precursors that spontaneously secrete antibodies related to autoimmune diseases [15] and further differentiate into plasma cells. Regulatory B cells (Breg), a subgroup of cells that negatively regulate immunity and inflammation, are considered closely related to the disorder of immune regulation in patients with DCM [16].

Alteration in the proportions of these peripheral B cell subpopulations are postulated to contribute to the pathogenesis of various autoimmune diseases, such as systemic lupus erythematosus (SLE), rheumatoid arthritis, multiple sclerosis and Sjogren’s syndrome. Moreover, the abnormal distribution is closely implicated in regulation of autoimmune disorders and the severity of these autoimmune diseases [17–19]. However, the distribution of B cell subpopulations in the peripheral circulation of patients with DCM has yet to be determined, and no report has described the association between B cell subsets and clinical features.

Thus, the purpose of this study was to prospectively profile the distribution of peripheral B cell subpopulations in patients with idiopathic DCM and determine their roles in the pathogenesis of DCM.

## Materials and methods

### Patients and healthy individuals

Twenty-seven patients with idiopathic DCM (DCM group), 18 control patients with heart failure (HF group) and 21 healthy controls (HC group) were included in this study. All subjects were recruited from the first affiliated Hospital of Guangxi Medical University.

The diagnosis of idiopathic dilated cardiomyopathy was based on the 1995 criteria from the WHO/ISFC and the proposed criteria from the ESC [2, 20]. After excluding secondary DCM, patients with a left ventricular end diastolic diameter (LVEDD) > 5.5 cm and left ventricular ejection fraction (LVEF) < 45% confirmed by echocardiography were included in the DCM group. The HF control group was established to minimize the potential confounding and bias caused by the aetiological heterogeneity of heart failure. Patients with heart failure caused by ischaemic cardiomyopathy, hypertensive heart disease and other diseases were enrolled in the HF group. Healthy individuals were defined as having no symptoms of heart failure and having a normal heart structure and function. Exclusion criteria included infections, malignant tumours, autoimmune diseases, rheumatism, diabetes, haematological diseases, endocrine diseases, severely impaired liver function and renal insufficiency. None of the subjects received immunosuppressive agents or were vaccinated in the two months prior to the study. This study is consistent with the principles outlined in the declaration of Helsinki and was approved by the Ethics Committee of first affiliated Hospital of Guangxi Medical University and obtained informed consent from all participants included in the study.

### Blood sample preparation

Peripheral fasting venous blood samples were collected in a heparin sodium anticoagulant vacutainer in the early morning. Part of the blood samples were centrifuged (1000 × g for ten minutes) and the plasma was collected and stored at -80 °C until analysis. Peripheral blood mononuclear cells (PBMCs) were isolated through Ficoll*-*Hypaque (Solarbio Technology Co., Ltd., Beijing, China) density gradient centrifugation (800 × g for twenty minutes). All blood samples were processed and cryopreserved within 3 h of collection.

### Flow cytometry

PBMCs were resuspended in phosphate-buffered saline (PBS) (Solarbio Technology Co., Ltd.) at a concentration of 2 × 10^6^ cells. PBMC suspensions (100 μl) were incubated with Fc receptor blocking reagent (BD Biosciences, San Jose, CA, no. 564219, 2.5 μg/test) at 4 ℃ for 10 min to inhibit nonspecific staining. All fluorescent dye-conjugated antibodies were pretitrated to obtain optimal results. The PBMCs suspension was then incubated with a fluorescent dye-conjugated antibody in the dark at 4 ℃ for 30 min for surface staining. The following fluorescent dye-conjugated antibodies were used: anti-CD3-APC-Cy7 (no. 557832, 1 μl/test), anti-CD19-APC (no. 555415, 5 μl/test), anti-CD20-PerCP-Cy5.5 (no. 560736, 1 μl/test), anti-CD27-PE-Cy7 (no. 560609, 1 μl/test), anti-CD43-PE (no. 560199, 5 μl/test), anti-CD38-BB515 (no. 564498, 1 μl/test), anti-CD5-APC-Cy7 (no. 563516, 1 μl/test), anti-CD24-PerCP-Cy5.5 (no. 561647, 1 μl/test), anti-IgD-PE (no. 555779, 8 μl/test) (all from BD Biosciences, San Jose, CA). Peripheral blood was analyzed using two panels specifically targeting B cell subsets. The first six-colour panel was designed for CD19, CD27, CD38, IgD, CD5 and CD24 antibodies to identify CD19^+^CD5^+^ B cells, CD19^+^CD24^hi^CD38^hi^ Breg cells, CD19^+^IgD^−^CD27^−^ double negative B cells (DN), CD19^+^IgD^+^CD27^+^ unswitched memory B cells (USwM), CD19^+^IgD^−^CD27^+^CD38^+^ switched memory B cells (SwM), CD19^+^IgD^−^CD38^hi^CD27^hi^ PB, CD19^+^IgD^+^CD27^−^CD38^−^CD24^+^ naïve B cells, CD19^+^IgD^+^CD27^−^CD38^+^CD24^lo^ mature B cells and CD19^+^IgD^+^CD27^−^CD38^hi^CD24^hi^ Tr cells. The second six-colour panel included CD3, CD19, CD20, CD27, CD43 and CD38 antibodies to distinguish CD3^−^CD19^+^D20^+^CD27^+^CD38^lo/int^CD43^+^ B1 cells. B Cell subsets were quantified according to their percentage among CD19^+^ lymphocytes.

**Fig. 1:**
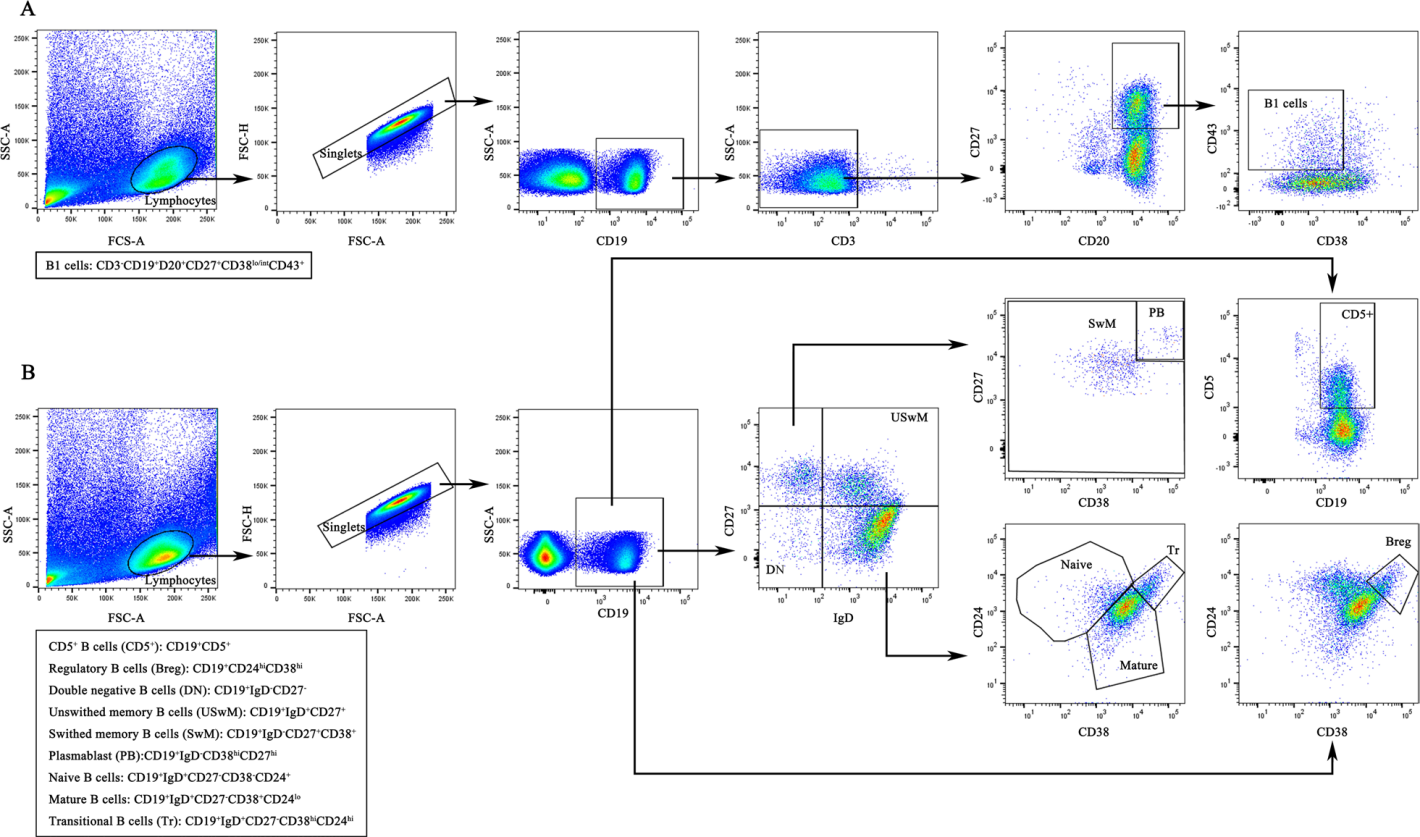
Gating strategy of two panels for identification of B cell subsets. A lymphocyte gate was selected in the forward scatter area (FSC-A)/side scatter area (SSC-A) scatter plots, then a singlet gate was strictly created in the FSC-A/forward scattering height (FSC-H) scatter plots. A total of 2 × 10^6^ events were collected within the singlet gate with low flow rate for analysis. **A** A six-colour panel included CD3, CD19, CD20, CD27, CD43 and CD38 antibodies to distinguish CD3^−^CD19^+^D20^+^CD27^+^CD38^lo/int^CD43^+^ B1 cells. **B** The other six-colour panel was designed for CD19, CD27, CD38, IgD, CD5 and CD24 antibodies to identify CD19^+^CD5^+^ B cells, CD19^+^CD24^hi^CD38^hi^ regulatory B cells (Breg), CD19^+^IgD^−^CD27^−^ double negative B cells (DN), CD19^+^IgD^+^CD27^+^ unswitched memory B cells (USwM), CD19^+^IgD^−^CD27^+^CD38^+^ switched memory B cells (SwM), CD19^+^IgD^−^CD38^hi^CD27^hi^ plasmablast (PB), CD19^+^IgD^+^CD27^−^CD38^−^CD24^+^ naïve B cells, CD19^+^IgD^+^CD27^−^CD38^+^CD24^lo^ mature B cells and CD19^+^IgD^+^CD27^−^CD38^hi^CD24^hi^ Transitional B cells (Tr)

After surface staining, PBMCs were washed with PBS and then resuspended in fixation buffer (1% paraformaldehyde). Finally, a flow cytometry analysis was performed with 488 nm and 640 nm lasers and 6 detectors using BD FACSVerse2L instrument (BD Biosciences). The gating strategy is shown in Fig. 1. A total of 2 × 10^6^ events were collected within the singlet gate with low flow rate. All data were analysed by FlowJo version 10 software (Tree Star Inc., Ashland, Oregon, USA). The compensation matrix was adjusted by single stained PBMCs and evaluated by fluorescence minus one.

### ELISA

After rewarming at 4 ℃ and room temperature, the plasma was assayed for IgM, IgG, IgG3, IL-10 and TNF-α levels according to the instructions of ELISA kits (Elabscience, Wuhan, China). The sensitivities were 1.88 ng/ml, 0.94 ng/ml, 0.1 ng/ml, 4.69 pg/ml and 4.69 pg/ml, respectively. The level of the β1-AR autoantibody was measured using ELISA kit (CUSABIO, Wuhan, China). An optical density (OD) sample/OD negative ratio < 2.1 was negative, while an OD sample/OD negative ratio ≥ 2.1was positive (if OD negative < 0.1, we calculated it as 0.1). All the samples were assayed in duplicate.

### Statistical analysis

The measurement data are presented as the means ± standard deviations. One-way ANOVA was used for comparisons among the three groups, and the LSD method was used for pairwise comparisons between groups. In addition, an independent sample *t test* was performed for comparisons between the two groups. Count data are presented as absolute numbers (percentages), and the chi-square test was used to compare the significant differences among the three groups. Pearson’s correlation analysis or Spearman’s rank correlation analysis was performed to determine correlations. *P* < 0.05 represented a significant difference. All data were analysed using SPSS version 26.0 software (SPSS, Inc., Chicago, IL, USA).

## Results

### Basic clinical characteristics and general blood parameters

The clinical baseline characteristics of the three groups are shown in Table 1. The sex, age, heart rate and serum creatine levels of the three groups were similar. Compared with the HC group, the DCM and HF groups had a higher NYHA functional classification, reduced LVEF and E/A ratio, enlarged LVEDD and elevated N-terminal pro-brain natriuretic peptide (NT-proBNP) levels. In addition, compared with the HF group and HC group, the mean arterial pressure (MAP) decreased and diastolic left ventricular posterior wall thickness (LVPWd) became thinner in the DCM group. In particular, compared with the HF group, the DCM group had a lower LVEF, larger LVEDD, thinner LVPWd and higher NYHA classification, but the E/A ratio was similar. Moreover, significantly increased high sensitivity C-reactive protein (hs-CRP) levels were measured in the HF group compared with the HC group.

**Table 1:** The basic clinical features

Characteristics	DCM (n = 27)	HF (n = 18)	HC (n = 21)	*P*
Sex (male/female)	24/3	17/1	19/2	0.82
Age (years)	55 ± 14	59 ± 13	50 ± 16	0.18
Disease duration (months)	49 ± 37^*^	64 ± 51^*^	–	0.00
BMI (kg/m^2^)	23.7 ± 4.4	24.6 ± 4.6	22.9 ± 4.1	0.48
Heart rate (beats/min)	78.5 ± 16.4	76.8 ± 15.9	73.6 ± 12.2	0.53
MAP (mmHg)	85.43 ± 3.21^*#^	89.67 ± 5.71	90.30 ± 4.13	0.00
Serum creatine (μmol/L)	94.57 ± 47.79	99.61 ± 44.32	79.34 ± 15.338	0.24
NYHA classification (II/III/IV)	14/7/6^*#^	1/10/7^*^	–	0.01
LVEF (%)	30 ± 6^*#^	41 ± 15^*^	67 ± 4	0.00
LVEDD (mm)	73 ± 8^*#^	65 ± 13^*^	50 ± 4	0.00
LVPWd (mm)	6.7 ± 0.8^*#^	9.2 ± 0.6	8.7 ± 0.6	0.00
E/A > 1 (n, %)	19,70.3%	13,72.2%	21,100%	0.02
NT-proBNP (pg/ml)	10,280.34 ± 1426.06^*#^	5880.32 ± 897.19^*^	411.49 ± 276.76	0.01
hs-CRP (mg/L)	4.378 ± 3.352	6.133 ± 3.816^*^	2.144 ± 2.541	0.01
Medications (n, %)				
ACEI/ARBs	15,55.6%^*^	12,66.7%^*^	–	0.00
β-blockers	19,70.4%^*^	10,55.6%^*^	–	0.00
Diuretics	24,88.9%^*^	13,72.2%^*^	–	0.30
Digitalis	21,77.8%^*^	8,44.4%^*^	–	0.02

The *P* value in the table represents the difference among the three groups, * represents a significant difference between DCM or HF and HC, and ^#^ represents a significant difference between DCM and HF. BMI: body mass index, MAP: mean arterial pressure, NYHA: New York Heart Association, CHD: coronary heart disease, LVEF: left ventricular ejection fraction, LVEDD: left ventricular end-diastolic diameter, LVPWd: diastolic left ventricular posterior wall thickness, Nt-proBNP: N-terminal pro-brain natriuretic peptide, hs-CRP: high sensitivity C-reactive protein, ACEI: angiotensin-converting enzyme inhibition, ARB: angiotensin receptor blockers

General blood parameters are listed in Table 2. Although the absolute number of lymphocytes in the DCM and HC groups was significantly lower than that in the HC group, the other blood parameters were similar among the three groups.

**Table 2:** General blood parameters

Parameters	DCM (n = 27)	HF (n = 18)	HC (n = 21)	*P*
Leukocytes (× 10^9^/L)	5.37 ± 2.93	6.04 ± 2.64	5.54 ± 2.46	0.71
Erythrocytes (× 10^12^/L)	5.76 ± 1.46	5.33 ± 1.73	5.62 ± 1.51	0.66
Hematocrit	0.43 ± 0.06	0.46 ± 0.06	0.44 ± 0.05	0.23
Hemoglobin (g/L)	146.92 ± 19.33	142.42 ± 16.11	148.73 ± 12.80	0.48
Lymphocytes (× 10^9^/L)	1.49 ± 0.52^*^	1.43 ± 0.65^*^	2.21 ± 0.84	0.00
Thrombocytes (× 10^9^/L)	209.43 ± 73.46	200.91 ± 71.63	213.31 ± 72.67	0.86

The *P* value in the table represents the difference among the three groups, * represents a significant difference between DCM or HF and HC

### Abnormal distribution of peripheral B1, Tr, USwM and SwM cell subsets in patients with DCM

We first analysed the percentage of CD19^+^ B and CD20^+^ B cells to investigate the difference in total B cell percentages among the three groups. No significant difference was observed in the percentage of CD19^+^ B cells (4.65 ± 4.00% for the DCM group vs. 6.04 ± 3.97% for the HF group vs. 5.67 ± 3.78% for the HC group, respectively, *P* = 0.46) (Additional file 1: Fig. S1 A, B) and CD20^+^ B cells (4.72 ± 3.42% vs. 6.61 ± 4.72% vs. 5.50 ± 2.76%, *P* = 0.24) among the three groups (Additional file 1: Fig. S1 C, D).

**Fig. 2:**
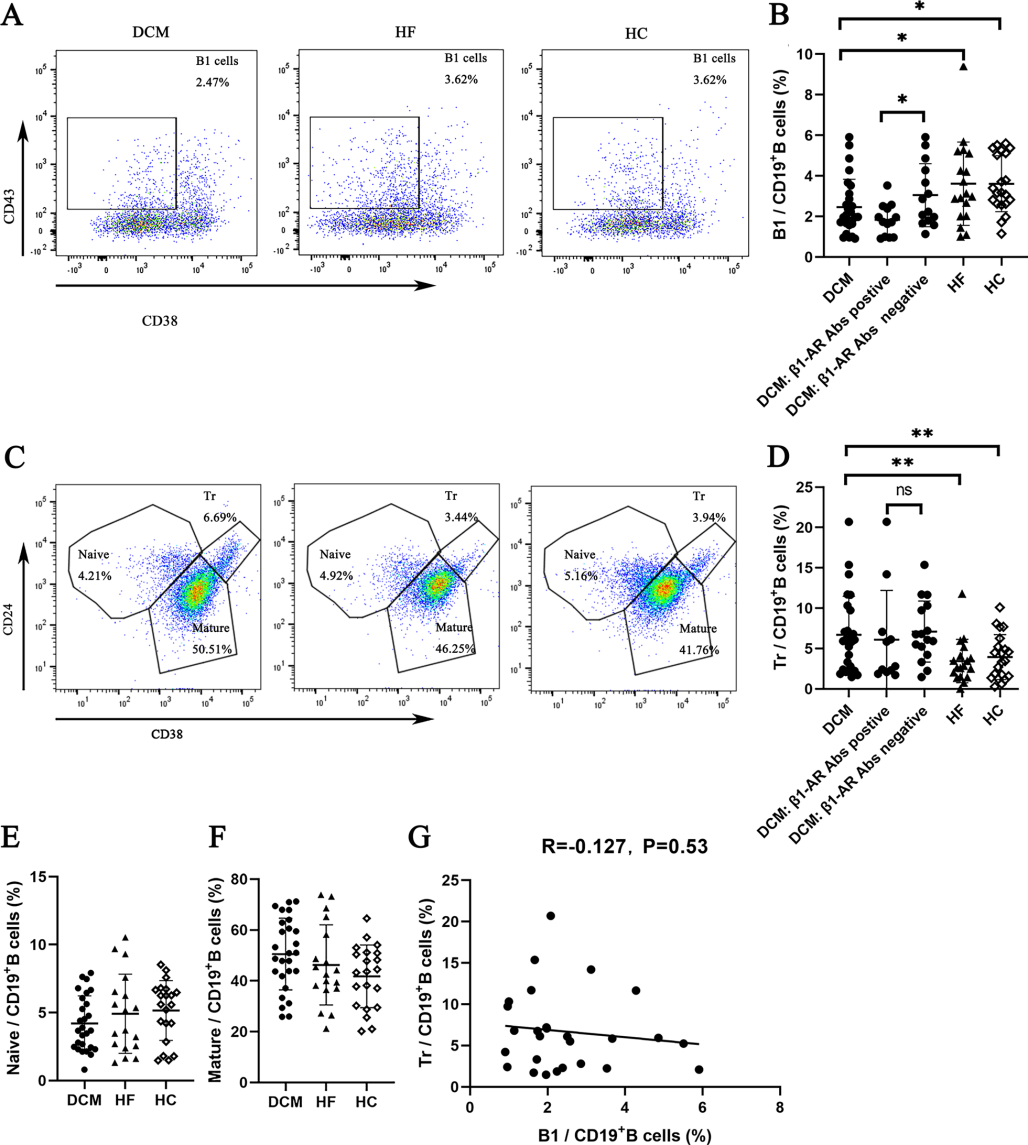
Comparison of B1, Tr, naïve and mature B cells among DCM, HF and HC groups. **A** Representative flow cytometry scatter plots of the percentage of B1 cells in the DCM (n = 27), HF (n = 18) and HC (n = 21) groups. The number in the figure represent the mean of the percentage of B1 cells in CD19^+^ B cells. **B** Comparison of the proportion of B1 cells in the peripheral blood among DCM, HF and HC groups. The results showed that the percentage of B1 cells in the DCM group was significantly lower than that in the HF group and HC group. Then, according to the expression of β1-AR autoantibodies, patients with DCM were divided into β1-AR autoantibody-positive and β1-AR autoantibody-negative subgroups, and the proportion of B1 cells was further compared. The results showed that the percentage of B1 cells in patients with DCM who were β1-AR autoantibody-positive was significantly lower than that in β1-AR autoantibody-negative patients. **C** Representative flow cytometry scatter plots of the percentages of Tr, naïve and mature cells among DCM, HF and HC groups. The number in the figure represent the mean of percentages of Tr, naïve and mature cells in CD19^+^ B cells. **D** Compared with the HF and HC groups, the percentage of Tr cells was significantly increased in the DCM group. However, the percentage of Tr cells was similar between β1-AR antibody-positive and β1-AR antibody-negative patients with HF. **E**, **F** No significant difference was detected in the percentages of naïve (**E**) and mature (**F**) B cells among the three groups. **G** No significant association was observed between B1 cells and Tr cells in patients with DCM. Each point represents an individual in figure B, D, E, F, G. Data were compared using one-way ANOVA or independent sample *t test*. *P < 0.05, **P < 0.01

**Fig. 3:**
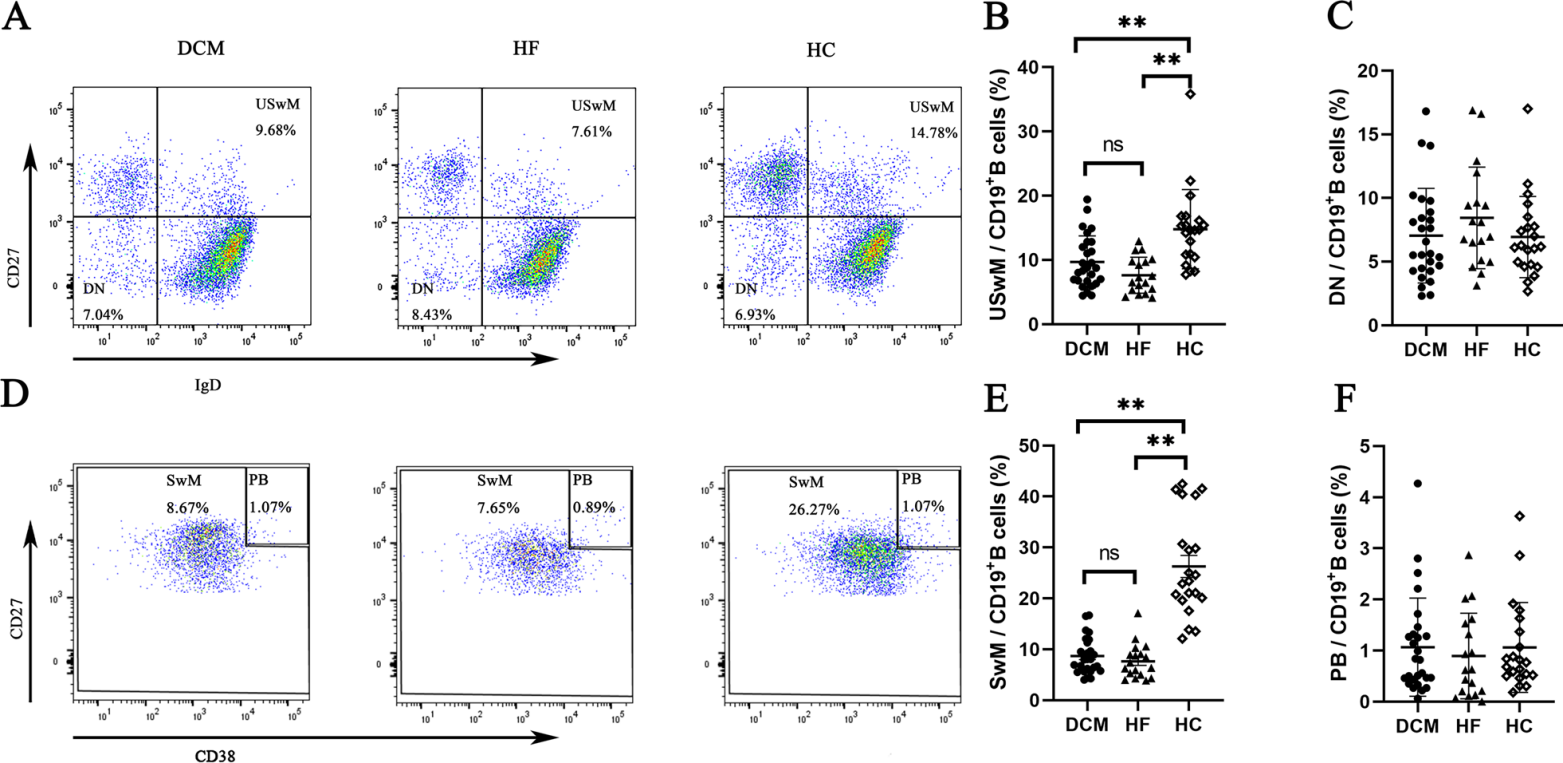
Comparison of USwM, SwM and PB cells among DCM, HF and HC groups. **A** Representative flow cytometry scatter plots of the percentages of USwM and DN cells in the DCM, HF and HC groups. The number in the figure represent the mean of percentages of USwM and DN cells in CD19^+^ B cells. **B** Compared with the HC groups, the percentage of USwM cells was significantly decreased in the DCM and HF groups. The percentage of USwM cells in the DCM and HF groups was similar. **C** The percentage of DN cells was similar among the three groups. **D** Representative flow cytometry scatter plots of the percentage of SwM and PB cells among DCM, HF and HC groups. The number in the figure represent the mean of percentages of SwM and PB cells in CD19^+^ B cells. **E** Compared with the HC groups, the percentage of SwM cells was significantly decreased in the DCM and HF groups. However, the percentage of SwM cells in the DCM and HF groups was similar. **F** The percentage of PB cells was similar among the three groups. Each point represents an individual in figure B, C, E and F. Data were compared using one-way ANOVA. **P < 0.01

Then, we analysed the distribution of B cell subpopulations in the three groups. Compared with the HF and HC groups, the percentage of B1 cells in the DCM group (2.47 ± 1.37% vs. 3.62 ± 2.05% vs. 3.62 ± 1.38%, *P* = 0.02) was significantly lower (Fig. 2A, B), whereas the percentage of Tr cells (6.69 ± 4.77% vs. 3.44 ± 2.69% vs. 3.94 ± 2.78%, *P* = 0.01) was significantly higher (Fig. 2C, E). However, no significant difference was observed in the percentage of B1 and Tr cells between the HF and HC groups. The percentages of USwM cells (9.68 ± 4.05% vs. 7.61 ± 2.79% vs. 14.78 ± 6.15%, *P* < 0.01) (Fig. 3A, B) and SwM cells (8.67 ± 3.53% vs. 7.65 ± 3.42% vs. 26.27 ± 9.88%, *P* < 0.01) (Fig. 3D, E) were significantly lower in the DCM and HF groups than in the HC group. Notably, the percentages of SwM and USwM cells in DCM and HF groups were similar. In addition, no significant differences in the percentages of naïve B cells (4.21 ± 2.02% vs. 4.92 ± 2.90% vs. 5.16 ± 2.20%, *P* = 0.35) (Fig. 2E), mature B cells (50.51 ± 14.10% vs. 46.25 ± 15.78% vs. 41.76 ± 12.31%, *P* = 0.12) (Fig. 2F), DN cells (7.04 ± 3.71% vs. 8.43 ± 4.00% vs. 6.93 ± 3.19%, *P* = 0.36) (Fig. 3C), PB (1.07 ± 0.96% vs. 0.89 ± 0.83% vs. 1.07 ± 0.88%, *P* = 0.35) (Fig. 3F), CD19^+^CD5^+^ B cells (8.53 ± 4.29% vs. 8.03 ± 5.68% vs. 11.73 ± 6.14%, *P* = 0.06) (Additional file 1: Fig. S2 A, B) and Breg (5.91 ± 5.65% vs. 3.71 ± 3.41% vs. 4.69 ± 2.99%, *P* = 0.25) (Additional file 1: Fig. S2 C, D) were observed among the three groups.

We divided patients with DCM into two subgroups with an LVEF 30%-45% and LVEF less than 30% to further determine the effect of cardiac function on B cell subsets. Although the proportion of B1 cells in the DCM subgroup with an LVEF less than 30% was lower than that in the subgroup with an LVEF > 30%, the difference was not significant (2.10 ± 1.25 vs. 2.88 ± 1.56, *P* = 0.18). In addition, significant differences were not observed in the percentages of CD19^+^ B cells (4.18 ± 3.35 vs.4.13 ± 2.91, *P* = 0.97), CD20^+^ B cells (4.60 ± 3.79 vs.4.83 ± 3.11, *P* = 0.87), Tr cells (6.80 ± 5.85 vs.6.20 ± 3.85, *P* = 0.76), USwM cells (10.28 ± 4.10 vs.9.94 ± 5.28, *P* = 0.85), SwM cells (9.24 ± 3.71 vs.8.90 ± 4.72, *P* = 0.84),naïve B cells (4.13 ± 2.07 vs.4.19 ± 2.18, *P* = 0.94), mature B cells (50.66 ± 15.43 vs.51.95 ± 14.85, *P* = 0.82), DN cells (8.47 ± 4.99 vs.6.33 ± 3.26, *P* = 0.20), PB (1.30 ± 1.21 vs.1.17 ± 1.33, *P* = 0.78), CD19^+^CD5^+^ B cells (7.65 ± 3.65 vs.8.74 ± 3.89, *P* = 0.49) and Breg cells (6.68 ± 6.50 vs.6.23 ± 6.23, *P* = 0.86) between the two subgroups (Additional file 1: Fig. S3).

Finally, we investigated the potential association between the percent changes in the populations of B1 cells and Tr cells. The correlation analysis did not reveal a significant correlation between the two cell types in patients with DCM (R = -0.127, P = 0.53) (Fig. 2G).

### Plasma levels of IgM, IgG and IgG3 were not affected by the abnormal distribution of B cell subsets in patients with DCM

**Fig. 4:**
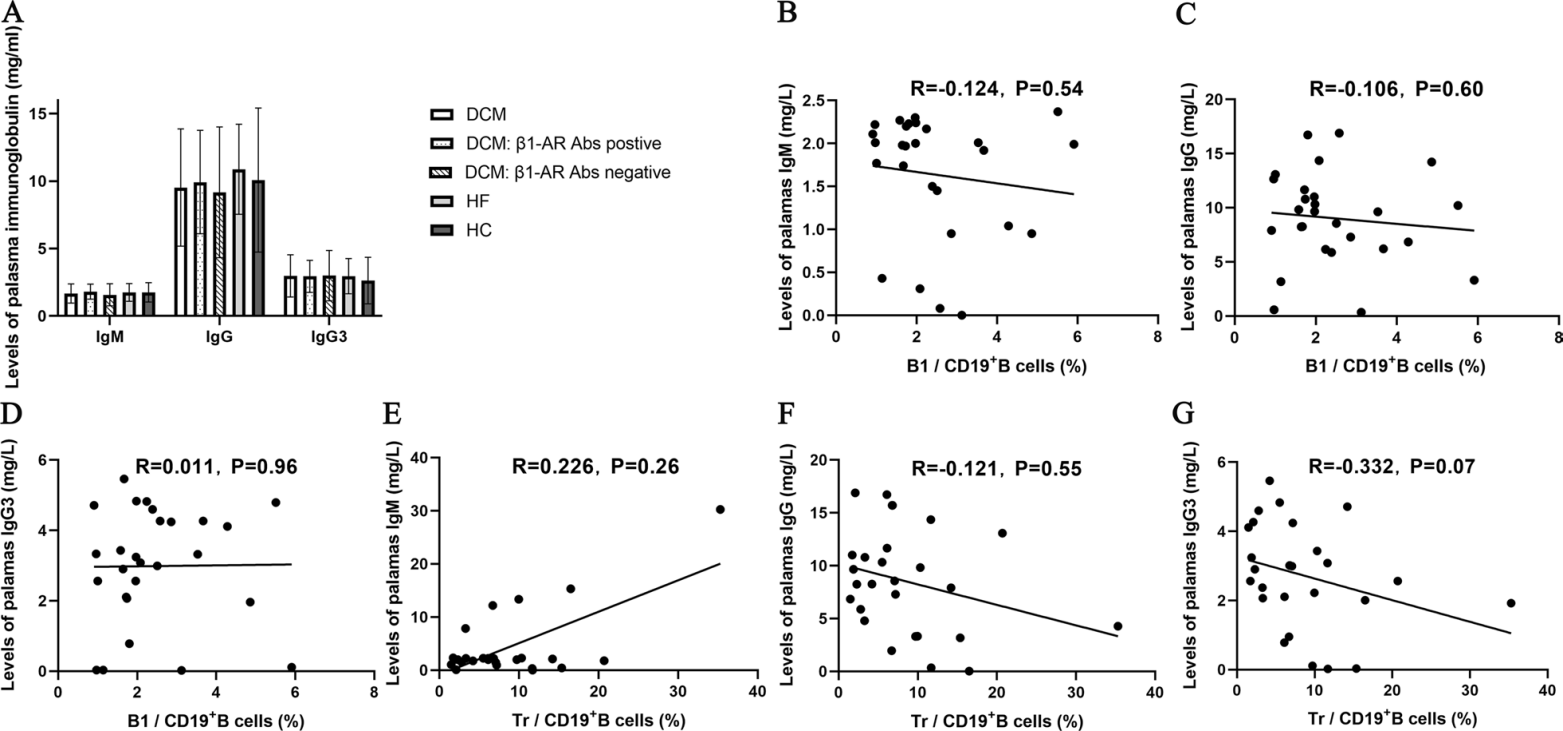
The effect of B1 and Tr cells on the plasma levels of immunoglobulin. The plasma IgM, IgG and IgG3 levels were detected by ELISA in the three groups. **A** The plasma levels of IgM, IgG and IgG3 were compared among DCM, HF and HC groups, and between patients with DCM who were β1-AR autoantibody-positive and h β1-AR autoantibody-negative. There was no significant difference in the levels of IgM, IgG and IgG3 among the three groups, and between the two DCM subgroups. Bars represent the means ± standard deviation. Data were compared using one-way ANOVA or independent sample *t test*. **B**–**D** The correlation between the percentage of B1 cells and the levels of plasma IgM (**B**), IgG (**C**) and IgG3 (**D**) in the DCM subgroup. **E**–**G** The correlation between the percentage of Tr cells and the levels of plasma IgM (**E**), IgG (**F**) and IgG3 (**G**) in the DCM subgroup. Neither the percentage of B1 cells nor the percentage of Tr cells was correlated with the levels of IgM, IgG and IgG3. Each point represents an individual in figure B-G. Pearson’s correlation analysis was performed

ELISAs were performed to determine the plasma IgM, IgG and IgG3 levels and determine the effect of the abnormal distribution of B cell subpopulations on plasma immunoglobulin levels in patients with DCM. However, no significant differences in the total plasma IgM (1.67 ± 0.71 vs. 1.75 ± 0.66 vs. 1.75 ± 0.72 mg/ml, *P* = 0.74), IgG (9.53 ± 4.35 vs. 10.88 ± 3.34 vs. 10.08 ± 5.34 mg/ml, *P* = 0.59) and IgG3 (2.97 ± 1.57 vs. 2.95 ± 1.31 vs. 2.63 ± 1.73 mg/ml, *P* = 0.72) levels were observed among the three groups (Fig. 4A).

Next, the correlations between the percentages of B1 cells and Tr cells and plasma immunoglobulin levels in patients with DCM were evaluated. The correlation analysis did not reveal significant correlations between B1 cells or Tr cells and plasma levels of IgM (R = -0.124, *P* = 0.54, for B1 cells; R = 0.226, *P* = 0.26, for Tr cells), IgG (R = -0.106, *P* = 0.60; and R = -0.121, *P* = 0.55, respectively) and IgG3 (R = 0.011, *P* = 0.96; and R = -0.332, *P* = 0.07, respectively) in patients with DCM (Fig. 4B–G).

### Plasma IL-10 and TNF-α levels were not affected by the abnormal distribution of B cell subsets in patients with DCM

**Fig. 5:**
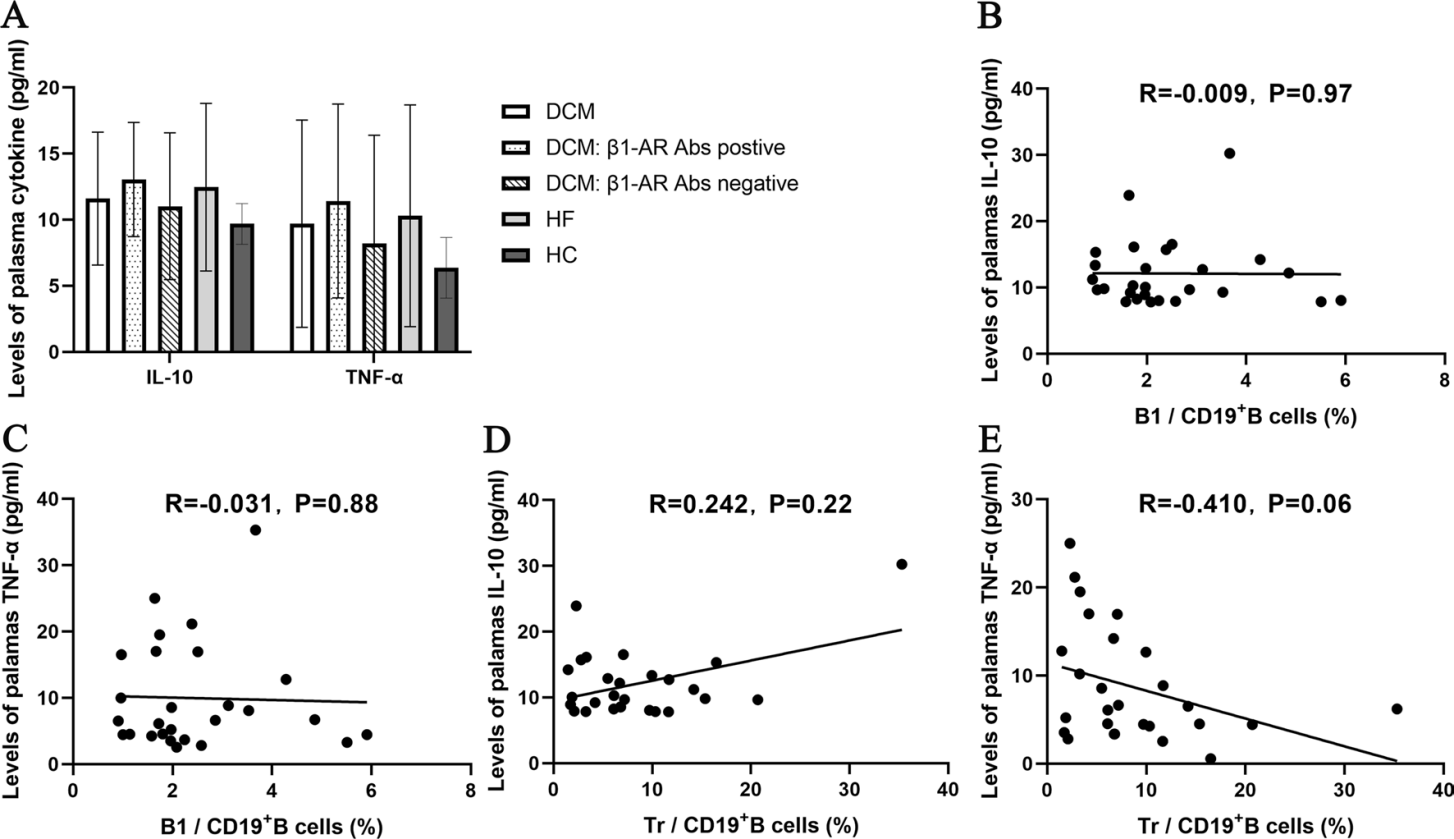
Effect of B1 and Tr cells on the plasma levels of IL-10 and TNF-α. The plasma IL-10 and TNF-α levels were assayed by ELISA in the three groups. **A** The levels of plasma IL-10 and TNF-α were analyzed among DCM, HF and HC groups, and between patients with DCM who were β1-AR autoantibody-positive and h β1-AR autoantibody-negative. No significant difference was found in the levels of plasma IL-10 and TNF-α among the three groups, and between the two DCM subgroups. Bars represent the means ± standard deviation. Data were compared using one-way ANOVA or independent sample *t test*. **B**, **C** The correlation between the percentage of B1 cells and the levels of plasma IL-10 and TNF-α in the DCM group. There was no significant correlation between the proportion of B1 cells and the levels of plasma IL-10 (**B**) and TNF-α (**C**) in the DCM group. **D**–**E** The correlation between the percentage of peripheral Tr cells and the levels of plasma IL-10 and TNF-α in the DCM group. No significant correlation was observed between the proportion of Tr cells and levels of IL-10 (**D**) and TNF-α (**E**). Each point represents an individual in figure B-E. Pearson’s correlation analysis was performed

We estimated the effect of altered proportions of B cell subsets on plasma levels of cytokines closely related to DCM by measuring the plasma levels of IL-10 and TNF-α using ELISA. Similar plasma levels of IL-10 (11.6 ± 5.03 vs. 12.47 ± 6.34 vs. 9.69 ± 1.54 pg/ml, *P* = 0.14) and TNF-α (9.70 ± 7.84 vs. 10.30 ± 8.39 vs. 6.37 ± 2.29 pg/ml, *P* = 0.14) were detected in the three groups (Fig. 5A).

Next, a correlation analysis was conducted to further explore the effect of changes in the B1 and Tr cell percentages on plasma IL-10 and TNF-α levels in patients with DCM. Neither B1 cells nor Tr cells were significantly correlated with plasma levels of IL-10 (R = -0.009, *P* = 0.97; R = 0.242, *P* = 0.22) and TNF-α (R = -0.031, *P* = 0.88; R = -0.410, *P* = 0.06) in patients with DCM (Fig. 5B–E).

### The percentage of B1 cells was significantly decreased in β1-AR autoantibody-positive patients with DCM

Levels of β1-AR autoantibodies in all subjects were detected using ELISA to evaluate the effects of β1-AR autoantibodies on the percentages of B1 and Tr cells and clinical parameters of patients with DCM. The positive rates of β1-AR autoantibodies in the DCM, HF and HC groups were 48.1%, 27.8% and 4.8%, respectively (*P* < 0.01). Then, we conducted a subgroup analysis of patients with DCM and HF according to the expression of β1-AR autoantibodies. In the DCM subgroup analysis, we found that the percentage of B1 cells in patients with DCM who were β1-AR autoantibody-positive was significantly lower than that in β1-AR autoantibody-negative patients (1.84 ± 0.78% vs. 3.05 ± 1.56%, *P* = 0.02) (Fig. 2B). However, similar results were not observed for Tr cells (6.10 ± 6.09% vs. 7.10 ± 3.78%, *P* = 0.61) (Fig. 2D). In addition, in the HF subgroup analysis, we did not detect a significant difference in the proportions of B1 and Tr cells (2.25 ± 0.94% vs. 3.79 ± 2.32%, *P* = 0.18; 2.30 ± 1.75% vs. 3.88 ± 2.90%, *P* = 0.28) between β1-AR antibody-positive and β1-AR antibody-negative patients with HF (Additional file 1: Fig. S4 A, B).

**Fig. 6:**
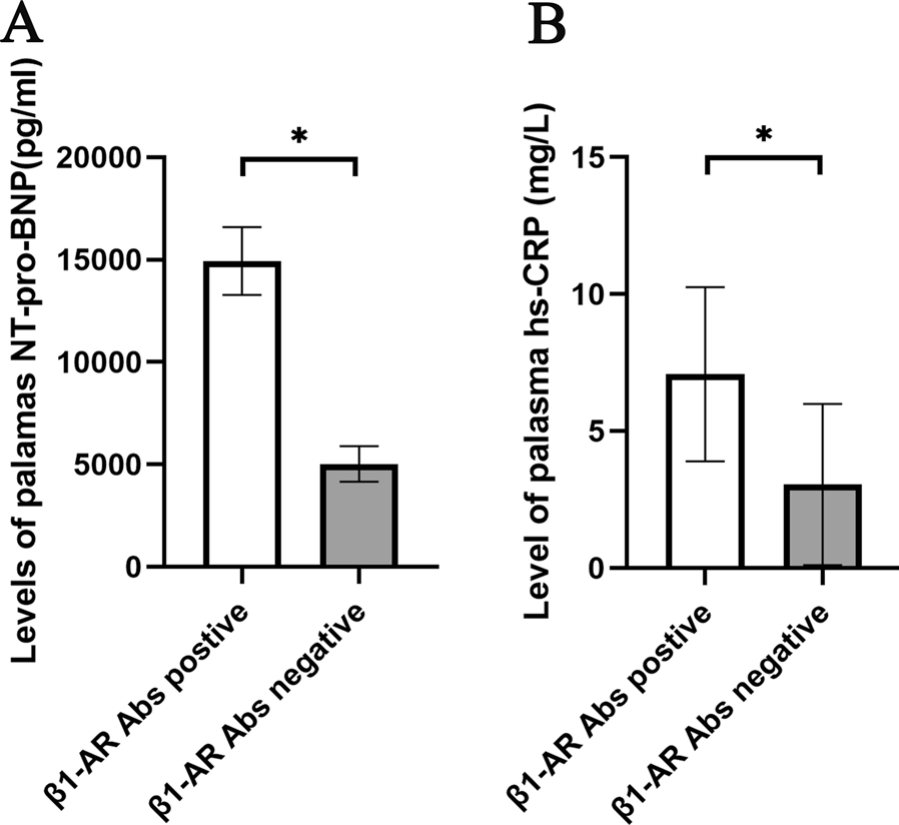
Effect of β1-AR autoantibodies on plasma NT-proBNP and hs-CRP levels in patients with DCM. **A**, **B** The levels of plasma NT-proBNP and hs-CRP were compared between in patients with DCM who were β1-AR autoantibody-positive and β1-AR autoantibody-negative. The plasma NT-proBNP (**A**) and hs-CRP (**B**) levels in patients with DCM who were β1-AR autoantibodies-positive were significantly higher than that in β1-AR autoantibody-negative patients. Bars represent the means ± standard deviation. Data were compared using independent sample *t test*. *P < 0.05

Interestingly, plasma NT-proBNP (14,932.09 ± 1655.47 vs. 5024.36 ± 863.16 pg/ml, *P* = 0.04) and hs-CRP (7.075 ± 3.187 vs. 3.049 ± 2.939 mg/L, *P* = 0.01) levels in patients with DCM who were positive for β1-AR autoantibodies were significantly higher than those in patients negative for β1-AR autoantibodies (Fig. 6). However, the plasma levels of IgM (1.80 ± 0.56 vs. 1.57 ± 0.82, *P* = 0.39), IgG (9.93 ± 3.83 vs. 9.17 ± 4.85, *P* = 0.64), IgG3 (2.94 ± 1.19 vs. 2.99 ± 1.87, *P* = 0.93), IL-10 (13.04 ± 4.32 vs. 11.02 ± 5.55, *P* = 0.28) and TNF-α (11.42 ± 7.33 vs. 8.20 ± 8.20, *P* = 0.27) were similar in the two subgroups (Figs. 4A, 5A).

We further clarified the relationship between beta-blockers, β1-AR autoantibodies and B1 cells by analysing the three variables in the DCM subgroup. The proportion of B1 cells was negatively correlated with β1-AR autoantibodies (R = -0.377, *P* = 0.02), but no correlation was observed between beta-blocker administration (R = 0.024, *P* = 0.88) and the B1 cell proportion and β1-AR autoantibodies (R = 0.042, *P* = 0.82).

### B1 cells were negatively correlated with NT-proBNP levels and positively correlated with the LVEF in patients with DCM

**Fig. 7:**
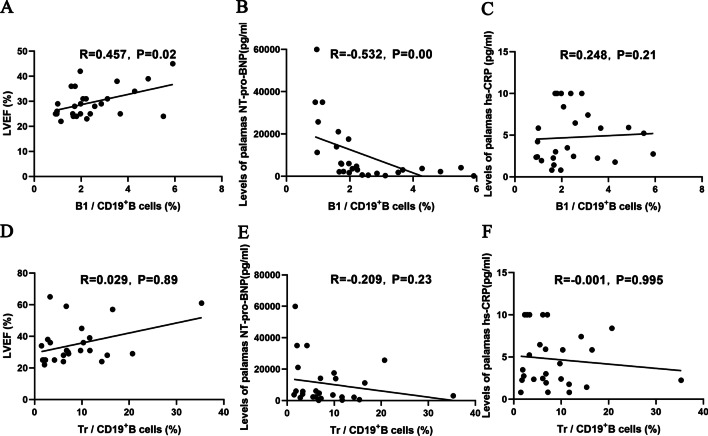
Correlation between the percentage of B1 and Tr cells and clinical features in patients with DCM. **A**–**C** The correlation analysis of the proportion of B1 cells with LVEF and the levels of plasma NT-Pro-BNP and hs-CRP levels. The percentage of B1 cells in the DCM group was positively correlated with the LVEF(A), negatively correlated with NT-proBNP levels (**B**) and not correlated with hs-CRP levels (**C**). **D**–**F** The correlation analysis of the percentage of Tr cells with the LVEF and the levels of plasma NT-Pro-BNP and hs-CRP. No significant correlation was found between the percentage of Tr cells and the LVEF (**D**), NT-pro-BNP (**E**) and hs-CRP levels (**F**). Each point represents an individual. Pearson’s correlation analysis was performed

A correlation analysis was performed to investigate the association between the percentages of B1 and Tr cells and the severity and prognosis of DCM. The percentage of B1 cells in the DCM group was negatively correlated with NT-proBNP levels (R = -0.532, *P* < 0.01), positively correlated with the LVEF (R = 0.457, *P* = 0.02), and not correlated with hs-CRP levels (R = 0.248, *P* = 0.21) (Fig. 7A–C). However, the correlations between the percentage of Tr cells and NT-proBNP levels (R = -0.209, *P* = 0.23), LVEF (R = 0.029, *P* = 0.89) and hs-CRP levels (R = -0.001, *P* = 0.99) were not significant (Fig. 7D–F).

Next, we further explored the role of the disease duration on the abnormal distribution of B1 cells and transitional B cells in the peripheral blood of patients with DCM. The results of the correlation analysis showed no significant correlations between the proportions of B1 cells (R = -0.198, *P* = 0.32) and Tr cells (R = 0.056, *P* = 0.78) and the disease duration in the DCM group (Additional file 1: Fig. S5 A, B).

## Discussion

In the present study, we used multicolour flow cytometry to reveal for the first time that the percentages of B1, USwM and SwM cells decreased and the percentage of Tr cells increased in patients with DCM compared with healthy individuals. The decrease in percentage of B1 cells in patients with plasma β1-AR autoantibody-positive DCM was more significant than that in patients with β1-AR autoantibody-negative DCM. The percentage of B1 cells in patients with DCM was negatively correlated with NT-proBNP levels and positively correlated with the LVEF.

B1 cells are unique cells involved in the immune response that play different roles in autoimmune diseases by producing immunoglobulins, presenting antigens to T cells and secreting immunomodulatory cytokines[8]. Different distributions of B1 cells in the peripheral circulation are observed in patients with various systemic immune diseases. The proportion of B1 cells is significantly increased in patients with SLE, and B1 cells stimulate the activation and proliferation of CD4^+^ T cells [21]. In contrast, the percentage of B1 cells decreases in patients with chronic graft-versus-host disease after allogeneic haematopoietic stem cell transplantation [22] and in patients with recurrent multiple sclerosis [17]. In the present study, the percentage of B1 cells decreased in patients with DCM, but the explanation for the decrease in B1 cells was unknown. In autoimmune arthritis and lupus mouse models, B1 cells migrate mainly to the spleen and inflammatory centres through signals generated by the CXCL13-CXCR5 axis [23, 24]. The decrease in the percentage of B1 cells in the peripheral blood of patients with multiple sclerosis is related to the increase in the level of CD11b molecules that regulate the adhesion and migration of B cells [17, 25]. Therefore, we speculated that the decrease in the percentage of B1 cells in peripheral blood of patients with DCM may be related to the redistribution of B1 cells into secondary lymphoid organs or tissues.

B1 cells mainly secrete natural IgM, but also secrete a small amount of IgG [15, 26]. The autoantibodies against the heart generated in patients with DCM are mainly IgG, especially IgG3. In addition, among many IL-10-secreting B cells, B1 cells are also one source of IL-10 [27]. The cytokine IL-10 exerts anti-inflammatory effects, negatively regulates immunity, and exerts a protective effect on the pathogenesis of DCM [28]. As shown in our previous study, the number of IL-10-producing B cells in the peripheral blood of patients with DCM increase, suggesting that these cells play an important role in the pathogenesis of DCM [29]. Since then, Jiao et al. [16] cocultured IL-10-producing B cells with helper T cells in vitro and further confirmed that these Breg cells significantly inhibit the secretion of TNF- α by helper T lymphocytes and thus play a protective role in DCM. In our study, the percentage of B1 cells in healthy individuals was approximately 2–5%. Due to the scarcity of B1 cells, we found that although the percentage of B1 cells decreased in patients with DCM, these cells did not exert a significant effect on plasma total IgM, IgG, IgG3, IL-10 and TNF-α levels.

Notably, we found that the decrease in the percentage of B1 cells was positively correlated with the decrease in the LVEF and negatively correlated with the increase in NT-proBNP levels. Generally, the LVEF decreases and NT-proBNP levels increase in patients with DCM, suggesting the occurrence of cardiac insufficiency and heart failure [30–32]. Therefore, the decrease in the B1 cell percentage in patients with DCM may be closely associated with the deterioration of cardiac function and heart failure. These observations suggest that the abnormal distribution of B1 cells may be involved in the pathogenesis of DCM.

Tr cells are intermediates in the development of mature human B cells. The stage of Tr cell differentiation is the key stage for the immune system to activate negative selection for the development of B lymphocytes. The deviation and abnormal proliferation of autoimmune Tr cells are considered the main causes of autoimmune diseases [33]. The number of Tr cells increase significantly in patients with autoimmune diseases such as multiple sclerosis, SLE and primary Sjogren's syndrome. The extent to which the proportion of Tr cells increases is related to the activity of SLE and primary Sjogren's syndrome, suggesting that an increase in the proportion of Tr cells in vivo directly or indirectly leads to the occurrence of autoimmune diseases [19, 34]. Consistent with previous studies [19, 34], our study reported an increase in the proportion of Tr cells in the peripheral blood of patients with DCM. However, the specific mechanism underlying the increase in the percentage of Tr cells in patients with DCM is unclear. According to previous studies, the main cause of the pathological increase in the number of Tr cells in peripheral blood is related to the release of a large number of Tr cells from bone marrow into circulation or the obstruction of migration to secondary lymphoid organs [35]. Notably, the correlation analysis showed that the percentage of Tr cells in the peripheral blood of patients with DCM was not related to the percentage of B1 cells, indicating that the increase in the percentage of Tr cells was not caused by the decrease in the percentage of B1 cells. Therefore, the increase in the percentage of Tr cells may be involved in the disorder of autoimmune regulation in patients with DCM.

Recent research clarifies that memory B cells are closely associated with autoimmune diseases mediated by antibodies. The decreased numbers SwM and USwM cells are negatively correlated with the disease activity of SLE and primary Sjogren's syndrome [19]. In our study, although we also found that the percentages of peripheral SwM and USwM cells were lower in patients with DCM than in healthy individuals, further comparison revealed similar decrease in the proportions of SwM and USwM cells in patients with DCM and patients with heart failure. These results suggest that the decrease in the percentages of SwM and USwM cells may be related to the cause of heart failure, and are not caused by DCM.

The production of anti-cardiac autoantibodies is caused by the loss of tolerance to cardiac self-antigens, which is an important hallmark of immune dysregulation in patients with DCM. Autoantibodies against β1-AR not only increase susceptibility to heart failure but also play a pathogenic role in myocardial remodelling by exerting sympathomimetic-like effects induced by binding to β1-AR [6]. Moreover, β1-AR autoantibodies promote cardiomyocyte apoptosis. Therefore, the appearance of β1-AR autoantibodies in the peripheral circulation is closely related to the increase in fatal ventricular arrhythmias, sudden death, and all-cause and cardiac mortality in patients with DCM [36–39]. In the present study, we found that the positive rate of β1-AR autoantibodies in patients with DCM was significantly higher than that in patients with heart failure and healthy subjects. Moreover, compared with β1-AR autoantibody- negative subjects, the percentage of B1 cells decreased more significantly in patients with DCM who were positive for β1-AR autoantibodies. The correlation analysis further confirmed that the proportion of B1 cells was negatively correlated with the level of β1-AR autoantibodies in patients with DCM. Based on these results, the decrease in the proportion of B1 cells in the peripheral blood of patients with DCM is closely related to the increased levels of β1-AR autoantibodies, indirectly suggesting that the decrease in the proportion of B1 cells in peripheral blood may increase the incidence of heart failure and mortality in patients with DCM.

Accumulating evidence confirms that B1 cells secrete polyclonal natural IgM antibodies that bind to a variety of immunogenic intracellular autoantigens expressed on the surface of apoptotic cells and accelerate the clearance of autoantigens by macrophages [12]. Once immune tolerance to apoptotic cells is disrupted, it will lead to the occurrence of a variety of chronic inflammation-related autoimmune diseases, including SLE, Sjogren's syndrome, and multiple sclerosis [11]. In the present study, we found that the proportion of B1 cells in patients with DCM was negatively correlated with the levels of β1-AR autoantibodies. Therefore, we speculated that with the decrease in the percentage of B1 cells in the peripheral blood of patients with DCM, the secretion of natural IgM also decreases, which affects the clearance of autoantibodies against apoptotic heart cells, which may explain the increase in β1-AR autoantibody levels in patients with DCM.

Although the mechanism by which a decrease in B1 cell numbers aggravates DCM is unclear, this pilot study reveals that decreased in the percentage of B1 cells are closely related to the decrease in the LVEF, the increase NT-proBNP levels and the increase in β1-AR autoantibody levels, indicating that decreased B1 cell numbers will exacerbate the deterioration of cardiac function and the progression of heart failure in patients with DCM. Therefore, the proportion of B1 cells in peripheral blood may be used as a potential biomarker to evaluate the severity of DCM.

## Conclusion

The present study documented an aberrant decrease in the percentage of B1 cells, especially in β1-AR autoantibody-positive patients, but a significant increase in the percentage of Tr cells in the peripheral blood of patients with idiopathic DCM, indicating that B1 cells and Tr cells may be involved in the pathogenesis of DCM. The decrease in percentage of B1 cells is directly related to the severity of DCM. Further studies of a larger sample are needed to verify these findings and to clarify the role of B cell subsets in the immune pathogenesis of DCM.

## Limitations

The principal limitations of this study are that it is a small-sample observational study, and only one point in the disease process was observed. Because the numbers of B1 and Tr cells in peripheral blood are very limited, sort B1 cells and Tr cells from patients with DCM are very difficult to sort for a functional evaluation. In addition, most of the subjects in this study were male, and thus these results may not be extrapolated to female patients. Larger cohorts of patients need to be recruited for further prospective studies to verify our findings and elucidate the pathophysiological functions and prognostic value of B cell subsets in patients with DCM.

## Supplementary Information

**Additional file 1: Fig. S1.** Comparison of CD19^+^ B cells and CD20^+^ B cells among DCM, HF and HC groups. **Fig. S2.** Comparison of CD5^+^ B cells and Breg cells among DCM, HF and HC groups. **Fig. S3.** Comparison of B cell subsets in patients with DCM with an LVEF 30%-45% and LVEF less than 30%. **Fig. S4.** Comparison of B1 cells and Tr cells in HF subgroups. **Fig. S5.** Correlation between the percentages of B1 and Tr cells and the disease duration in patients with DCM.

## Data Availability

All data generated or analyzed in this study are included in this paper.

## Abbreviations

DCM: Dilated cardiomyopathy
β1-AR: β1 Adrenergic receptor
NT-proBNP: N-terminal pro-brain natriuretic peptide
LVEF: Left ventricular ejection fraction
Tr: Transitional B cells
USwM: Unswitched memory B cells
SwM: Switched memory B cells
TNF-α: Tumor necrosis factor alpha
Breg: Regulatory B cells
PB: Plasmablast
IL-10: Interleukin-10
SLE: Systemic lupus erythematosus
LVEDD: Left ventricular end diastolic diameter
PBMCs: Peripheral blood mononuclear cells
PBS: Phosphate-buffered saline
OD: Optical density
MAP: Mean arterial pressure
LVPWd: Diastolic left ventricular posterior wall thickness
hs-CRP: High sensitivity C-reactive protein
DN: Double negative B cells
