# Supplementary material for: The abnormal distribution of peripheral B1 cells and transition B cells in patients with idiopathic dilated cardiomyopathy: a pilot study
Source: BMC Cardiovasc Disord. 2022 Mar 4;22:78. doi: 10.1186/s12872-022-02461-8 (PMC8895850; doi:10.1186/s12872-022-02461-8)
Supplement: Supplementary file 1 — Additional file 1: Fig. S1. Comparison of CD19+ B cells and CD20+ B cells among DCM, HF and HC groups. Fig. S2. Comparison of CD5+ B cells and Breg cells among DCM, HF and HC groups. Fig. S3. Comparison of B cell subsets in patients with DCM with an LVEF 30%-45% and LVEF less than 30%. Fig. S4. Comparison of B1 cells and Tr cells in HF subgroups. Fig. S5. Correlation between the percentages of B1 and Tr cells and the disease duration in patients with DCM. [file 12872_2022_2461_MOESM1_ESM.docx]

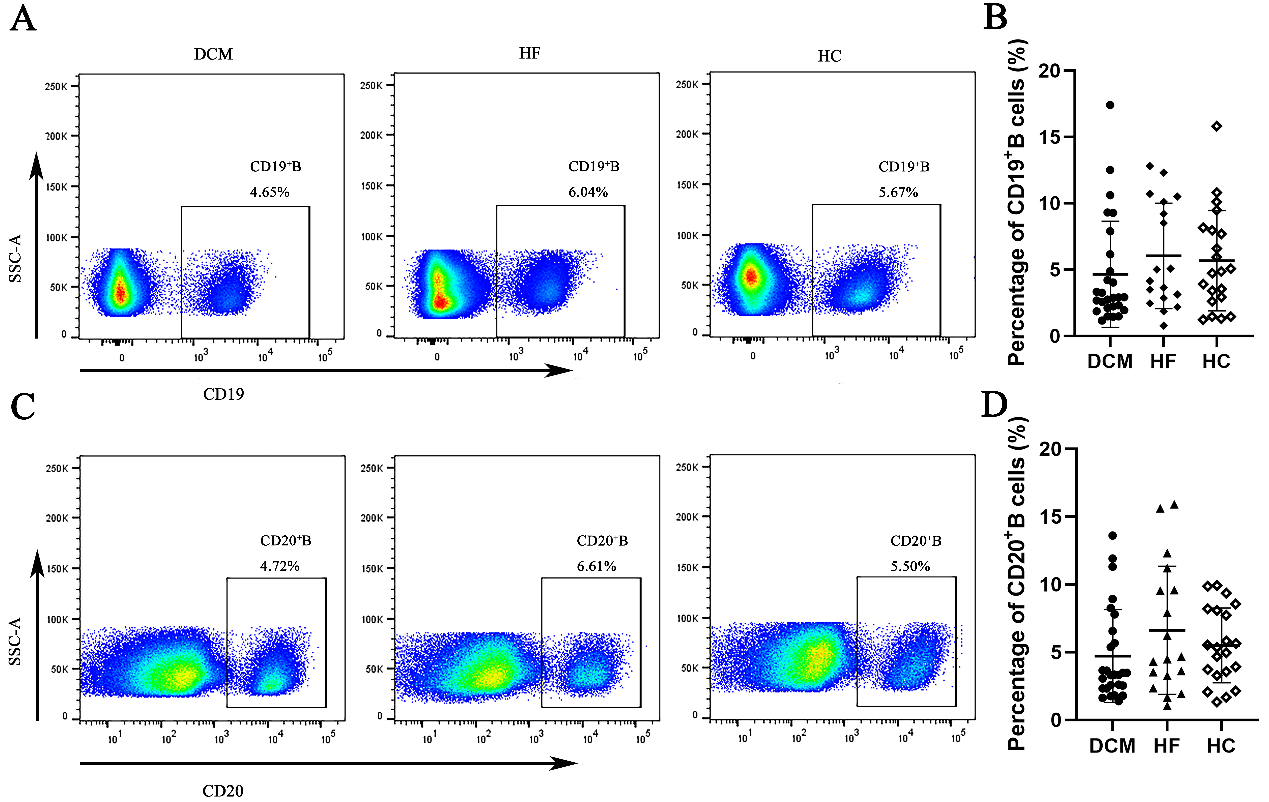


**Fig. S1 Comparison of CD19^+^ B cells and CD20^+^ B cells among DCM, HF and HC groups.** (A) Representative flow cytometry scatter plots of the percentage of CD19^+^ B cells among DCM (n=27), HF (n=18) and HC (n=21) groups. The number in the figure represent the mean of percentage of CD19^+^ B cells. (B) No significant difference was observed in the percentage of total CD19^+^ B cells among the three groups. (C) Representative flow cytometry scatter plots of the percentage of CD20^+^ B cells among the three groups. The number in the figure represent the mean of percentage of CD20^+^ B cells. (D) The DCM, HF and HC groups had a similar the percentage of CD20^+^ B cells. Each point represents an individual in figure B and D. Data were compared using one-way ANOVA.


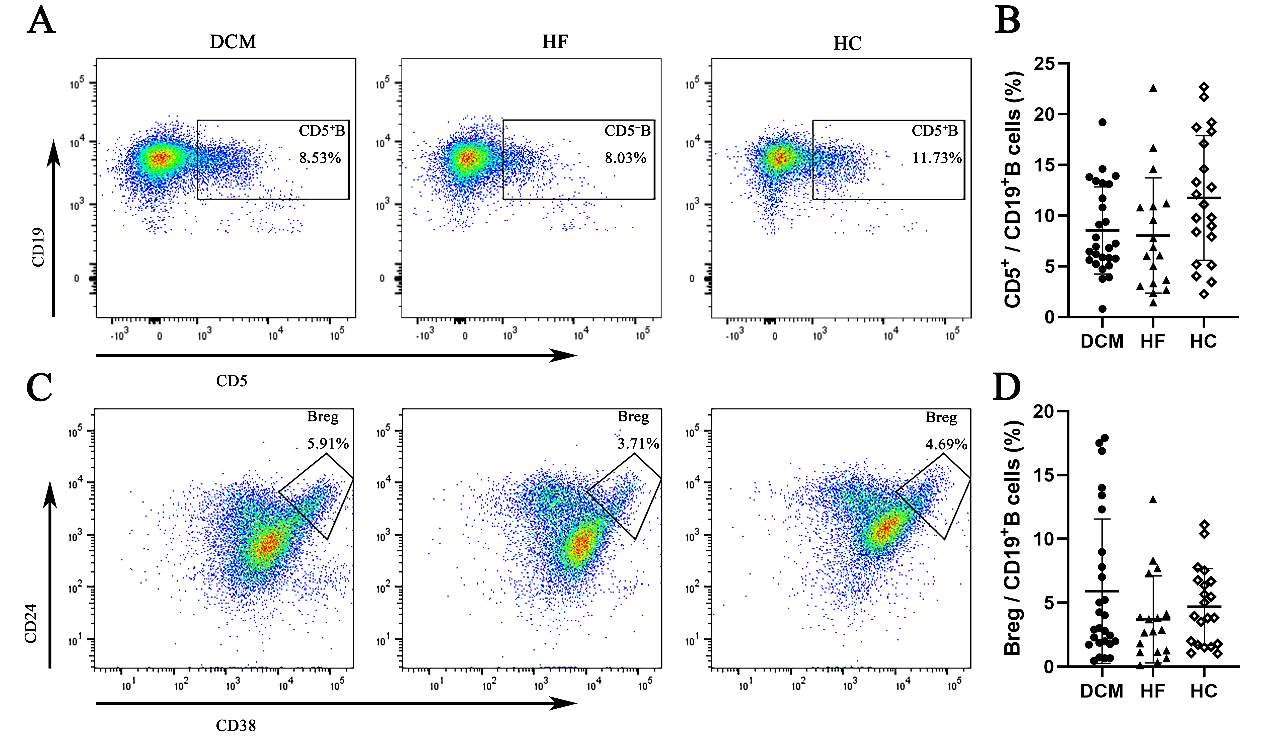


**Fig. S2 Comparison of CD5^+^ B cells and Breg cells among DCM, HF and HC groups.** (A) Representative flow cytometry scatter plots of the percentage of CD5^+^ B cells among DCM, HF and HC groups. The number in the figure represent the mean of percentage of CD5^+^ B cells in CD19^+^ B cells. (B) No significant difference was found in the percentage of CD5^+^B cells among the three groups. (C) Representative flow cytometry scatter plots of the percentage of Breg cells among the three groups. The number in the figure represent the mean of percentage of Breg cells in CD19^+^ B cells. (D) The DCM, HF and HC groups had a similar the percentage of Breg cells. Each point represents an individual in figure B and D. Data were compared using one-way ANOVA.

**
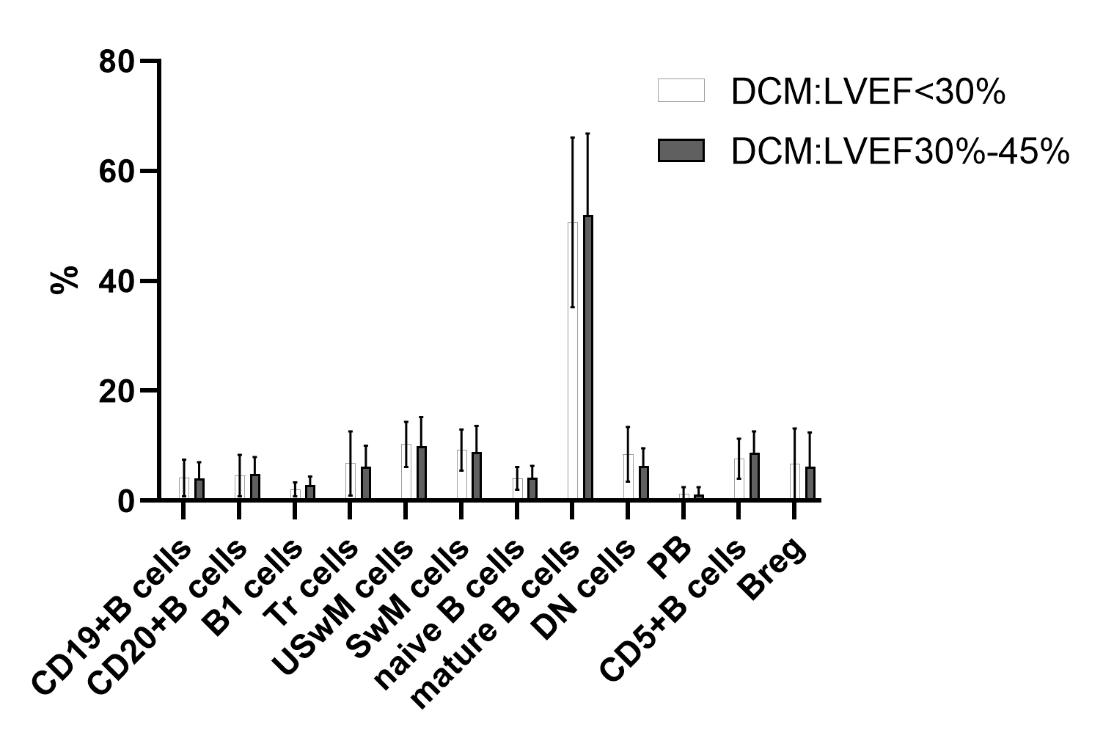
**

**Fig. S3 Comparison of B cell subsets in patients with DCM with an LVEF 30%-45% and LVEF less than 30%.** There was no significant difference in the percentages of CD19^+^ B cells, CD20^+^ B cells, B1 cells, Tr cells, USwM cells, SwM cells, naïve B cells, mature B cells, DN cells, PB, CD19^+^CD5^+^ B cells and Breg between the two subgroups. Data were compared using independent sample *t test*.


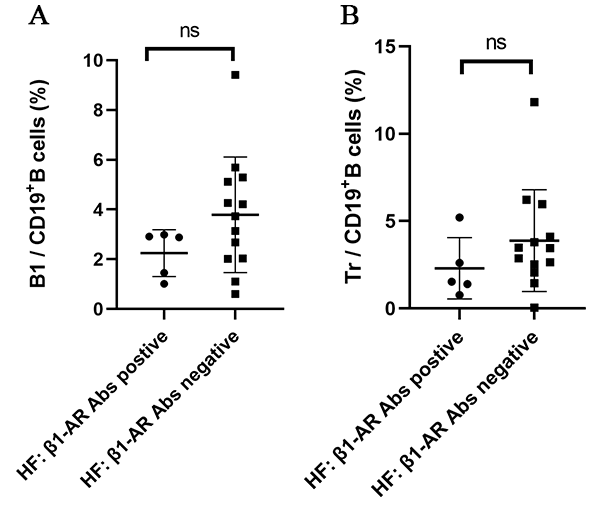


**Fig. S4 Comparison of B1 cells and Tr cells in HF subgroups.** (A) Comparison of B1 cells between β1-AR antibody-positive (n=5) and β1-AR antibody-negative (n=13) patients with HF. (B) Comparison of Tr cells between β1-AR antibody-positive (n=5) and β1-AR antibody-negative (n=13) patients with HF. The results showed that there was no significant difference in the proportions of B1 and Tr cells between the two subgroups. Data were compared using independent sample *t test*.


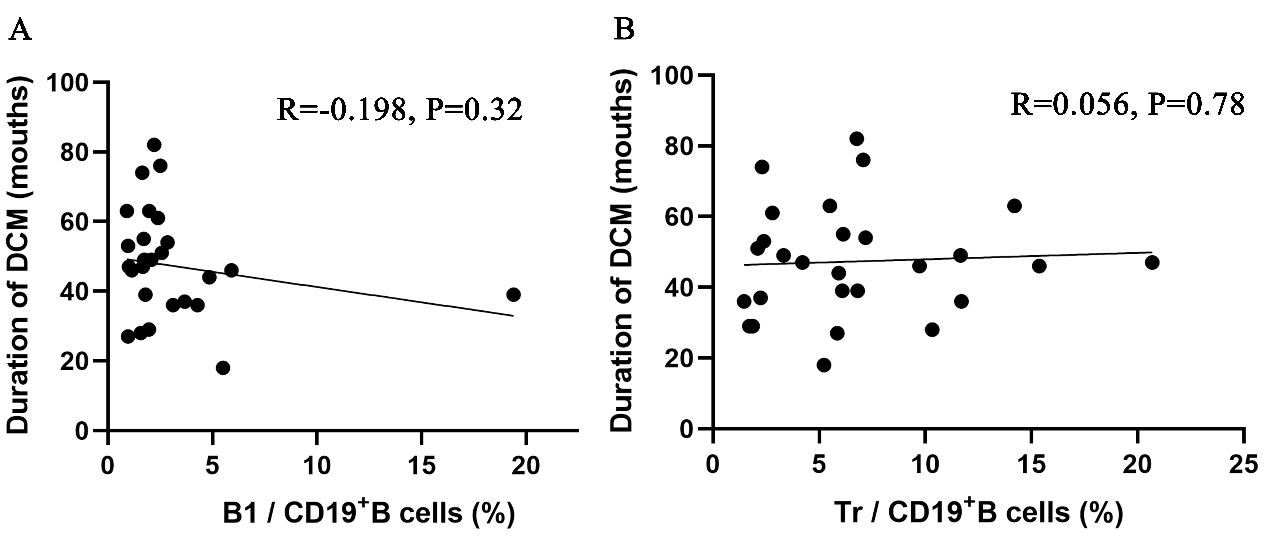


**Fig. S5 Correlation between the percentages of B1 and Tr cells and the disease duration in patients with DCM.** There was no significant correlation between the proportions of B1 cells (A) and Tr cells (B) and the disease duration in the DCM group. Each point represents an individual. Pearson’s correlation analysis was performed.
